# Polyandry is shaped by male mating behavior in house mice living in semi-natural conditions

**DOI:** 10.1093/beheco/arag084

**Published:** 2026-08-06

**Authors:** Fragkiskos Darmis, Anja Guenther

**Affiliations:** Research Group Behavioural Ecology of Individual Differences, Max Planck Institute for Evolutionary Biology, August-Thienemann-Straße 2, Plön 24306, Germany; Research Group Behavioural Ecology of Individual Differences, Max Planck Institute for Evolutionary Biology, August-Thienemann-Straße 2, Plön 24306, Germany; Department of Zoology and Animal Ecology, University of Hildesheim, Universitätsplatz 1, Hildesheim 31141, Germany

**Keywords:** alternative reproductive tactics, environmental variation, fitness, multiple paternity, polyandry, social environment

## Abstract

Polyandry is widespread across animals, yet its underlying causes and consequences remain unclear in natural populations. Recent work suggests that spatial and temporal variation in the environment shape the causes and consequences of polyandry. Here, we extend this research by testing whether one consequence of polyandry, multiple paternity, is influenced by females’ social environment, specifically the prevalence of male alternative reproductive tactics (ARTs), territorials and roamers. We studied semi-natural populations of house mice (*Mus musculus domesticus*) under different food quality treatments. Doing so allowed us to explore whether the relationship between male ARTs and multiple paternity depends on environmental conditions, which are expected to mediate the relative costs and benefits of reproductive investment. Using microsatellite genotyping, we assigned paternity to 329 litters produced by 257 females across 5 generations. We then classified the sires of these litters as territorials or roamers based on their reproductive behavior around conception and quantified each sire's reproductive contribution to each litter. Multiple paternity was strongly associated with the presence of roamers, which were twice as likely to occur in multiple-sired litters compared with territorials. Territorials still sired most offspring, an effect consistent with roamers engaging in sneaky/opportunistic matings. However, we found no clear evidence that environmental conditions shift how ARTs contribute to multiple paternity. Together, our results demonstrate a tight link between female mating outcomes and male ARTs across environments, implying that the drivers of polyandry depend on the social environment. By explicitly linking ARTs to multiple paternity, our study provides a mechanistic understanding on how social dynamics shape reproductive outcomes in mating systems where both sexes mate multiply.

## Teaser

In house mice, males use different mating strategies—some defend territories, while others roam to sneak matings—and these tactics influence paternity. We found that roaming males increased the chance of litters having multiple fathers. This shows how social dynamics shape reproduction, highlighting that the benefits of multiple mating depend on the male mating landscape.

## Introduction

Mating with more than one partner, once thought to be a male characteristic only (Tang-Martínez 2016), is now recognized as widespread in females across species and taxa (Arnqvist and Nilsson 2000; Kvarnemo and Simmons 2013; Parker and Birkhead 2013; Pizzari and Wedell 2013; Fromonteil et al. 2023). However, this behavior presents a key challenge in behavioral ecology: what are the drivers and consequences of polyandrous mating? Most research addressing this question focuses on the fitness consequences of polyandry, with adaptive hypotheses’ main prediction being that offspring resulting from polyandrous mating increase (some component) of female fitness (Pizzari and Wedell 2013; Brouwer and Griffith 2019).

The majority of studies on polyandry are on socially monogamous systems, such as birds, where females have a social partner but nevertheless mate and produce clutches with multiple fathers (ie show extra-pair paternity: Schlicht and Kempenaers 2011; Forstmeier et al. 2014; Maldonado-Chaparro et al. 2018; Brouwer and Griffith 2019). There, polyandry has been shown to confer fitness benefits, for example by increasing the heterozygosity of females’ offspring (Foerster et al. 2003). In polygynandrous species, where both males and females mate multiply, polyandry can result in multiple paternity when more than 1 male sires offspring within a litter (Clutton-Brock and Isvaran 2006). The frequency of multiple paternity varies substantially across species. For example, among rodents (Solomon and Keane 2008), the proportion of multiple-sired litters ranges from ∼29% in house mice (Thonhauser, Thoß, et al. 2014) to 90% in the yellow-toothed cavy (Hohoff et al. 2003). Such pronounced variation suggests that the costs and benefits of polyandry differ across ecological and social contexts. Indeed, polyandry and the resulting multiple paternity have been associated with reduced infanticide risks (Klemme and Ylönen 2010), inbreeding avoidance (Firman and Simmons 2008a) or good genes via sperm competition (Kvarnemo and Simmons 2013), among others.

Explaining why these benefits vary so markedly across species and populations remains a major challenge. First, it is unclear what are the benefits of polyandry for males and females simultaneously. Second, who drives multiple mating (Eliassen and Kokko 2008), ie males coercing females (Clutton-brock and Parker 1995), female choice, females facilitating (convenience) polyandry (Huchard et al. 2011) or interactive effects among male and female traits (Ramos et al. 2014), is notoriously hard to show in wild populations (Taylor et al. 2014; Fromonteil et al. 2023; Darmis and Guenther 2025; Yan et al. 2026). Third, even within the same species, the incidence of multiple mating varies substantially among populations (Dean et al. 2006; Firman and Simmons 2008a; Firman and Simmons 2008b; Thonhauser et al. 2013; Forstmeier et al. 2014; Thonhauser, Raveh, et al. 2014; Maldonado-Chaparro et al. 2018), suggesting that fine-scale variation in social and environmental conditions may shape its occurrence. Last, the central prediction of adaptive hypotheses, that multiple mating provides fitness benefits to females, is not always supported (Forstmeier et al. 2014; Ortega et al. 2023). These results suggest that either multiple mating is not always associated with fitness benefits or that its fitness consequences are context-dependent, emerging from interactions between behavior and variation in social and environmental conditions across space and time (Forstmeier et al. 2014; Maldonado-Chaparro et al. 2018; Darmis and Guenther 2025).

An unexplored hypothesis that accounts for this spatial and temporal variation in male and female behavior in respect to mating links polyandry with the frequency of male alternative reproductive tactics (ARTs hereafter), ie the occurrence of distinct mating behaviors when males pursue matings (Gross 1996; Brockmann 2001; Wolff 2008). These tactics often include territorial and roamer males. Territorials defend areas where females aggregate, typically because they are more competitive (eg are heavier or more aggressive), while roamers, which are generally less competitive, adopt an opportunistic tactic by moving between territories in search of sneaky matings with unguarded females. Variation in the relative frequency of these tactics could directly influence the incidence of polyandry. Roamers might steal matings and/or coerce females, something too costly for females to resist (Huchard et al. 2011). They may also increase infanticide risk owing to their limited mating opportunities, which will elevate the incidence of polyandry if females mate multiply in an attempt to confuse paternity and reduce this risk (Blaffer Hrdy 1974; Klemme and Ylönen 2010; Forstmeier et al. 2014). Conversely, territorial males may also actively contribute to multiple paternity by stealing fertilizations from neighboring males. Indeed, in striped mice *Rhabdomys pumilio* (Schradin et al. 2010; Schradin and Lindholm 2011), territorials are more aggressive toward neighbors, which steal most paternity, while in banded mongooses, *Mungos mungo*, vocalizations and inspections increase more in response to scent marks from neighbors than from strangers (Müller and Manser 2007), suggesting heightened neighbor-directed reproductive or territorial interactions.

Importantly, the interaction between male and female mating behavior cannot be understood independently of environmental conditions. Environmental conditions, such as resource availability or quality, can shape population density, competitive interactions and the spatial distribution of individuals, thereby influencing both the relative frequency of territorial and roaming males, as well as the distribution of females across space. Because ARTs are often maintained through condition-, density- or frequency-dependent processes (Gross 1996; Brockmann 2001; Iserbyt et al. 2013; Darmis et al. 2025), shifts in environmental conditions can alter the costs and benefits associated with each tactic, and consequently their prevalence within populations. In turn, these changes are likely to affect female mating behavior if roaming males increase coercion or infanticide risk under harsher conditions, when territorial males are expected to mate-guard more intensively. Environmental conditions are an increasingly recognized mediator of the consequences of female behavior too, especially of polyandry (Hoi-Leitner et al. 1999; Say et al. 1999; Schmoll et al. 2005; Arct et al. 2013; Darmis and Guenther 2025), because they shape offspring survival and potentially the maternal investment in litters (Darmis and Guenther 2025). Overall, environmental conditions are expected to determine the extent to which variation in male and female behavior translates into realized reproductive success. Particularly, environmental quality may play a key role, because it influences the energetic resources available for investment into reproduction (Reznick et al. 2000).

Here, we explore an intriguing hypothesis connecting all the above, if multiple paternity in house mice (*Mus musculus domesticus*) is linked to male ARTs as a function of environmental quality. House mice are ideal for this because their social structure is characterized by a polygynous deme formation (Drickamer 2001): females have multiple-sired litters (Firman and Simmons 2008a; Thonhauser et al. 2013) and breed year-round, while males switch between territoriality and roaming mostly based on their competitive ability (Darmis et al. 2025), but nevertheless display some consistency in their choice of tactic. In replicated semi-natural populations that differed only in the quality of the food provided, we first estimated whether polyandry is influenced by an aspect of females’ social environment, the number of males following a territorial and a roaming tactic around conception. The social environment is considered a key mediator of polyandry and its potential benefits (Cohas and Allainé 2009; Maldonado-Chaparro et al. 2018) and we know that the frequency of territorials and roamers varies over time (Darmis et al. 2025). We therefore expected multiple paternity to increase with the relative abundance of roamers if they sneak matings. Moreover, we expected this relationship to be weaker in lower quality environments. In such environments, where reproduction is more constrained, litter sizes are smaller and competition is therefore higher (Prabh et al. 2023; Darmis and Guenther 2025), roamers may increase efforts to steal paternity. Alternatively, territorials might also intensify mate guarding, thereby reducing cuckoldry and multiple paternity. Second, we explored which tactic contributes most offspring in multiple-sired litters by quantifying the tactic of the fathers around the conception of the litter. Last, we quantified how many offspring each father (and hence ARTs) contributed to each litter. We expected roamers to sire a disproportionate share of offspring within multiple-sired litters primarily in high-quality environments, where larger litter sizes and higher female reproductive output (Prabh et al. 2023) increase roamers’ opportunities for successful fertilizations.

## Materials and methods

### Animals

For our analyses we used female mice (*N* = 257) that lived freely in 4 semi-natural enclosures of 19.6 m^2^ each (Prabh et al. 2023; Darmis et al. 2025). We focused on females that had at least one litter with more than 1 pup surviving to be sampled for genetic analyses (*N*_observations_ = 329 from these 257 females).

Wild house mice were first captured in the Cologne/Bonn region of Germany. Descendants of 18 wild-caught breeding pairs were used to establish 4 replicate semi-natural enclosures, each containing 20 males and 20 females (160 individuals in total). The enclosures ran continuously from 2019 to 2024, encompassing overlapping generations, were predator-free, closed with a roof and designed to mimic the natural conditions in which house mice form colonies, in barns (König and Lindholm 2012). Specifically, within each enclosure, food and water were distributed across 9 stations and replenished daily to be ad libitum, simulating a barn environment where food is abundant (Laurie 1946; Berry 1982; König and Lindholm 2012). Enclosures were exposed to natural daylight and temperature fluctuations, with underfloor heating maintaining a minimum of 10 °C as burrowing was not possible. Bedding and 13 nest boxes provided nesting material and space. Under these conditions, mice in our enclosures reproduce throughout the year, with peak reproductive activity occurring between April and September.

Two of the 4 enclosures (enclosures 1 and 2) always received standard-quality food Altromin 1324 (SQ) and the 2 others (enclosures 3 and 4) always high-quality food Altromin 1414 (HQ). The main difference between the 2 food qualities is the amount of energy available per gram of food. The standard-quality food contained 3,227 kcal/kg metabolizable energy (24% from protein, 11% from fat and 65% from carbohydrates) while the high-quality food contained 3,680 kcal/kg (28% from protein, 22% from fat and 50% from carbohydrates). Consequently, the standard-quality food is of lower nutritional and energetic quality. Previous research has indicated the importance of the food quality and of the diet for the reproduction and behavior of house mice (Bronson 1979; Prabh et al. 2023; Darmis and Guenther 2026). On average, animals fed high-quality food reproduce faster, have shorter generation times (Prabh et al. 2023) and are more risk-averse compared with animals fed standard-quality food (Lopez-Hervas et al. 2026), which need to eat more throughout 24 h even in a non-reproductive state (Prabh et al. 2023). Overall, our replicated semi-natural enclosure system standardizes other major environmental variables (eg space availability, overall habitat structure) and allows us to robustly estimate survival and population size. Thus, food quality represents the primary manipulated ecological axis that creates variation across populations.

Of the 257 females we analyzed, 167 were housed in enclosures 1 and 2 and 162 in enclosures 3 and 4. For all analyses, we grouped enclosures 1 and 2 together as the standard-quality food ones, and enclosures 3 and 4 together as the high-quality food ones. We combined observations of enclosures with the same food quality to increase our sample size and statistical power, an approach justified because the only systematic difference between the enclosures is food quality. However, to account for random variability among enclosures, enclosure identity (1 to 4) was included as a random intercept in all models (see Statistical analyses).

After releasing the founders, we monitored population developments every 4 to 5 wk (ie conducted full population checks). During these monthly censuses, we caught all mice within an enclosure to measure their body mass, took a tissue sample (ear clip; DNA sample) of new individuals (weighing >10 g) to assign maternity and paternity using microsatellite analysis, and implanted new animals with an RFID PIT tag (Planet ID, 1.4 × 9 mm) for recognition. All handling and sampling were performed on site, and no local or general anesthesia was used during ear clipping. This procedure is standard in small mammal field studies and was approved (see Ethics section). Following sampling, individuals were placed in standard cages until all animals of each enclosure had been processed, after which they were released back into their respective enclosures.

As exceeding the enclosure carrying capacity (140 adults, Krebs et al. 2019) can negatively affect survival, particularly of juveniles, we removed the older generation once the number of RFID-tagged offspring exceeded 80 (corresponding to a total of 160 individuals, including adults and subadults). This removal typically occurred every 8 months (range 6 to 10). Importantly, because of these removals, observations were restricted to a maximum female age of ∼12 months, which nonetheless represents the upper end of the reproductive lifespan in this species (Bellamy et al. 1973; Morgan and Bellamy 1975). In other words, all removed individuals (ie from the preceding/older generation) had nearly completed their “reproductive lifespan” (Darmis et al. 2025; Darmis and Guenther 2025). Because of these removal events and the natural fluctuations in density, population sizes per enclosure varied between 39 and 200 individuals, with a mean of ∼96.

### Assigning parentage and multiple paternity

We assigned parentage using 17 microsatellite markers from DNA extracted from ear clips (Linnenbrink et al. 2013). Samples were amplified with QIAGEN Multiplex PCR kits and run on an ABI 3730 Sequencer (Applied Biosystems). Alleles were called in GeneMarker (V2.6.4), and parentage was assigned using Colony (v2.0.7.2) based on the maximum likelihood of each potential parental pair (Jones and Wang 2010). An individual was accepted as an assigned parent if the estimated likelihood was 95% or higher.

We grouped pups into litters for each mother. We linked the genetic data with census records and identified the first date each pup received a RFID PIT tag (>10 g, corresponding to ∼30 d old). Pups of the same mother tagged during the same census were assigned to the same litter. For example, if a female produced 6 offspring over her lifespan, and 4 offspring of the same age were tagged during 1 session while 2 additional pups—also of the same age as each other but different from the first 4—were tagged in a later session, we assigned these to 2 separate litters. Then, we determined multiple paternity within each litter: a litter was classified as multiple sired if pups within that litter were assigned to different fathers based on the microsatellite parentage results (eg a litter of 5 pups, 3 sired by male A and 2 by male B, was considered multiple sired).

Importantly, our estimates of litter size and multiple paternity reflect the genetic sampling of ∼10 g old pups during censuses, ie roughly of 2 wk of age. In other words, we could not reliably estimate the early-life survival of pups sired by different male tactics. Most pup mortality is thought to occur within the first few days after birth (1 to 2 d), often before systematic detection is possible. In addition, females may transfer pups between nest boxes, further complicating accurate tracking of early-life survival. Consequently, we are unable to assess whether early mortality differs between offspring sired by roamers versus territorials, consistent with limitations in comparable semi-natural and wild population studies (König and Lindholm 2012; Darmis and Guenther 2025).

### Variables quantified

We estimated the date of conception for each litter based on the timing of offspring tagging. Young mice are tagged at 10 g, corresponding to an age of ∼10 to 16 d. Given a gestation period of 20 to 21 d, conception was estimated to have occurred roughly 1 mo (4 to 5 wk) prior to tagging. To quantify the number of roamers and territorials at the time of conception, we assigned reproductive tactics to adult males (ie individuals more than ∼1-month post-chipping). Each male was classified as either territorial (T) or roamer (R) at the estimated time of conception but could switch between reproductive events (Darmis et al. 2025). Territorial males were defined as those found alone in a nest box or as the heaviest male present in a nest box (smaller/lighter males are usually the subadult offspring of the territory holder) around conception. Roamers included all other adult males present in the population. These differences reflect meaningful variation in how males move in enclosures and how much time they spent in nest boxes (Darmis et al. 2025; Bandyopadhyay et al. 2026). Importantly, because the number of nest boxes is limited per enclosure (*n* = 13), only a subset of males can establish and defend territories, constraining the expression of territoriality.

Importantly, the operational sex ratio (OSR), defined as the number of adult males relative to sexually receptive (ie non-pregnant, non-lactating females) females (Emlen and Oring 1977), ranged from 0.26 to 4.20 across enclosures, with a mean of ∼1.1. Given that the OSR was, on average, close to 1:1 and followed a comparable distribution across all 4 semi-natural enclosures, its effect on mating opportunities is likely limited in our system. Moreover, because the number of territories (nest boxes) is fixed, variation in the number of males is primarily driven by the number of non-territorial individuals (roamers). Indeed, the number of roamers is strongly correlated with the number of adult males (Spearman's *ρ* = 0.94, *P* < 0.0001), whereas the number of territorial males shows only a weak but significant association with adult male numbers (*ρ* = 0.11, *P* < 0.0001). For these reasons, we did not consider the OSR in our analyses.

### Statistical analyses

All analyses were performed in R 4.2.1 in a frequentist framework using the *lme4* package (Bates et al. 2015). For categorical predictors, we used dummy contrasts as implemented by default in R. Validity of all models was assessed with residual plots and by checking collinearity among predictors in the *performance* package (Lüdecke et al. 2021). All numeric variables were standardized (mean = 0, SD = 1) to aid model convergence and facilitate interpretation. For all analyses, we included only litters with more than 1 surviving pup, as litters with a single pup cannot, by definition, exhibit multiple paternity.

We first tested whether multiple paternity was associated with the abundance of male ARTs around the conception of litters, quantified as the absolute number of roamers and of territorials. We further examined whether this relationship differed as a function of food quality in the environment. To this end, we fitted a binomial generalized linear mixed-effects model (GLMM) with litter paternity status (single versus multiple sired) as the response. The number of roamers and of territorials were included as predictors, each interacting with food quality. Mother and enclosure identity (1 to 4) were included as random intercepts to account for non-independence among litters from the same female and for random noise among enclosures. Litter size and food quality were further included as interacting covariates to control for the higher detectability of multiple paternity in larger litters and for the influence of food quality on litter size (Prabh et al. 2023).

We then explored whether territorials or roamers sire the most offspring in multiple-sired litters to infer which tactic contributes most. To do so, we restructured the dataset so that each row represented a unique father–litter combination, allowing litters with multiple sires to appear multiple times (*N* = 245 fathers, *N* = 329 litters). Only litters with more than 1 surviving pup were considered. Each father was classified as “territorial” or “roamer” based on its spatial position during the census around conception, following the same procedure described above. Thus, a litter with 2 fathers contributed 2 observations: 1 per father and his corresponding tactic (*N*_total_ = 454 observations). Litters for which any father's tactic was unknown were excluded to increase the robustness of our results. For each father–litter combination in this newly created dataset, we assigned the number of genotyped offspring sired by that father on that litter. Overall, for each litter combination we had the identity of the fathers of the pups, their tactic around conception and how many offspring each father sired in that litter. Consequently, we built a Poisson GLMM with the reproductive success of each father as the response. We included father tactic around conception, multiple paternity of the litter (whether the litter was multiple sired or not) and food quality as interacting predictors (3-way interaction) to capture context-dependent effects: whether the reproductive success of roamers versus territorials depended on whether litters were single or multiple sired and on environmental quality. Random intercepts for father, litter and enclosure identity were initially included to account for repeated measures of the same males, litters and enclosures; however, they contributed zero variance (singular fits) and were therefore excluded from the final model. This indicates that variation in offspring number among repeated observations was largely explained by the fixed effects. After fitting the model, we used the *emmeans* package to run post hoc tests on interactions with 2 (or more) categorical factors (eg litter status × father tactic × food quality).

Last, we aimed to determine which male tactic contributed most frequently to multiple paternity, since both territorial males and roamers can potentially do so. To achieve this, we focused on litters with more than 1 father and assigned, within each litter, the father with the largest number of genotyped offspring as the “main father.” Fathers contributing fewer offspring than the main father were classified as “contributors” to paternity. Only territorial males could be assigned as main fathers since roamers, by definition, gain paternity through an alternative, non-dominant behavior. Litters in which multiple fathers had an equal number of offspring were excluded because we could not reliably assign any father as the “main father.” Using this classification, we quantified for all contributors the number of litters in which they sired pups and their tactic around conception. In total, we analyzed 126 observations from 74 litters and 99 unique fathers across several generations. We then calculated the proportion of contributors that were roamers and territorials and statistically tested whether this proportion deviated from an equal expectation of 0.5 using a 1-sample proportion test.

## Results

Our dataset contained a total of 329 litters with more than 1 surviving pup that were confidently assigned as single or multiple sired. Of these, 108 were multiple sired (33%). Importantly, we could assign paternity in 320 of these 329 litters, meaning that for some multiple-sired litters one (or more) of the fathers could not be identified. Litter size was at an average of 4.02 ± 2.06 pups (range = 2 to 15). Roamers fathered offspring in more litters than territorials (264 times fathers were roamers; 157 unique individuals versus 181 times fathers were territorials; 105 unique individuals), a statistically significant difference (*χ*^2^ = 10, df = 1, *P* = 0.0013).

**Figure 1:**
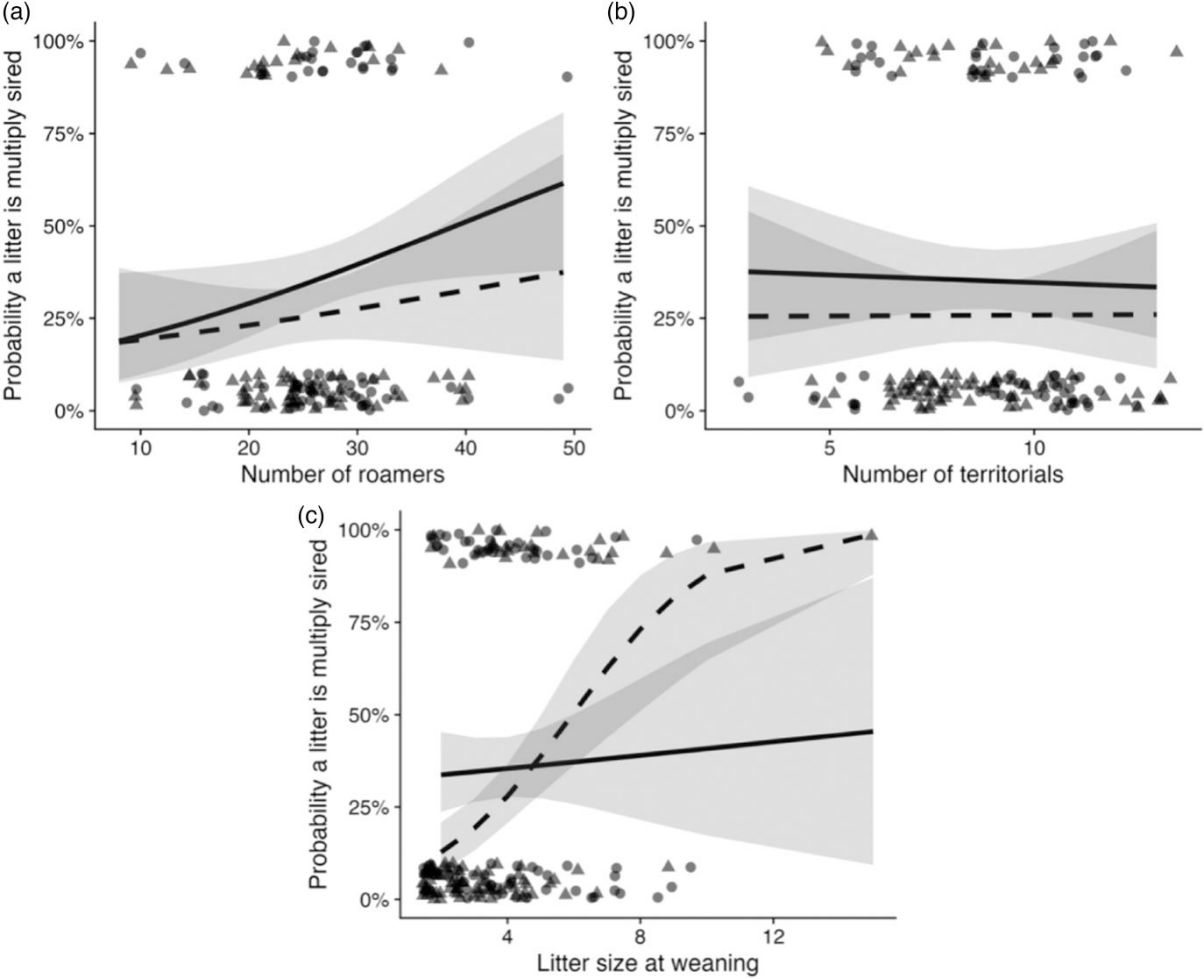
The probability of multiple paternity as a function of the (a) number of roaming males and (b) number of territorial males. Solid lines and circles represent the high-quality food enclosure; dashed lines and triangles represent the standard-quality one; (c) and litter size per food quality treatment enclosure. Lines show predicted probabilities from the generalized linear mixed model with 95% Cis (shaded areas). Raw data points are jittered for visualization. Importantly, excluding the one observation with litter size = 15 (upper right of figure c) did not change neither the direction nor the significance of any estimate (results not shown).

The probability of multiple paternity significantly increased with an increasing number of roamers around conception (*β* = 0.37, SE = 0.18, *P* = 0.039; Fig. 1a; Table 1), something not detected for territorial males (*β* = −0.04, SE = 0.17, *P* = 0.8; Fig. 1b; Table 1). Moreover, there was no significant interaction between tactic and food quality (roamers × food: *β* = 0.83, SE = 0.24, *P* = 0.5; Table 1). Importantly, larger litters were more likely to be multiple sired in the standard-quality enclosures only (litter size × food: *β* = 0.87, SE = 0.29, *P* = 0.003; Fig. 1c; Table 1).

**Table 1:** Results of the 2 models we used in this study.

Predictors	Model 1			Model 2		
	Odds ratios	CI	*P*	Incidence rate ratios	CI	*P*
Intercept	−0.61	−0.98–−0.23	**0.001**	1.38	1.23–1.51	**<0.001**
No. of roamers	0.37	0.02–0.73	**0.039**			
Standard-quality food enclosures	−0.43	−0.98–0.12	0.122	−0.23	−0.42–−0.04	**0.017**
No. of territorials	−0.04	−0.36–0.29	0.820			
Litter size	0.07	−0.29–0.44	0.696			
No. of roamers × standard-quality food enclosures	−0.19	−0.74–0.37	0.513			
No. of territorials × standard-quality food enclosures	0.04	−0.51–0.59	0.877			
Litter size × standard-quality food enclosures	0.87	0.30–1.43	**0.003**			
Territorial father				−0.08	−0.28–0.13	0.458
Multiple-sired litter				−0.93	−1.15 –−0.71	**<0.001**
Multiple-sired litter × territorial father				0.40	0.07–0.73	**0.017**
Standard-quality food enclosures × territorial father				0.18	−0.11–0.47	0.220
Multiple-sired litter × standard-quality food enclosures				0.48	0.16–0.80	**0.003**
Multiple-sired litter × standard-quality food enclosures × territorial father				−0.23	−0.70–0.25	0.344

Model 1 tested whether the probability that a litter is multiple sired depends on the number of roamers or of territorials around conception as a function of food quality in the environment. Model 2 tested if the number of pups sired in each litter by each male is predicted by the interaction between male tactic, multiple paternity and food quality in the environment. Significant results at a level <0.05 are highlighted in bold.

**Figure 2:**
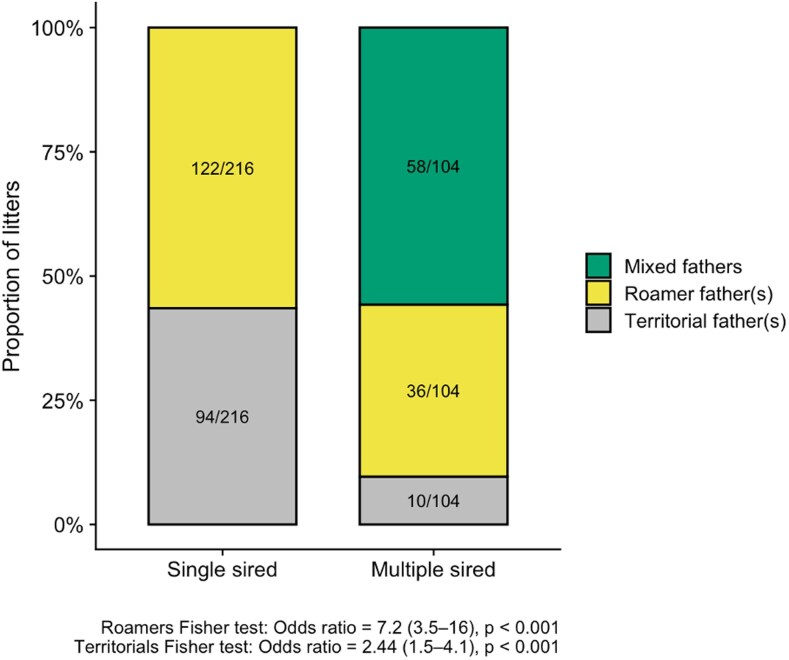
Composition of father types in single- and multiple-sired litters. Stacked bars show the proportion of litters containing only roamer fathers, only territorial fathers, or a mixture of both tactics. Values inside bars indicate the number of litters in each category out of the total per litter type. Multiple-sired litters were more likely to contain both roamer and territorial males compared with single-sired litters. Fisher’s exact tests indicate significant differences in the presence of roamer fathers (odds ratio = 7.2, 95% CI = 3.5 to 16.4, *P* < 0.001) and territorial fathers (odds ratio = 2.4, 95% CI = 1.5 to 4.1, *P* < 0.001) between litter types.

Among litters where the tactics of all fathers could be assigned with statistical confidence (*N* = 320), 104 were multiple sired and 216 were single sired. Roamers were present in more than 90% (94/104) of multiple sired and in 56% (122/216) single-sired ones (ie in those 122 only roamers contributed pups). Consequently, a Fisher's exact test indicated that multiple-sired litters were significantly more likely to include (at least) one roamer than single-sired ones (odds ratio = 7.2, 95% confidence interval [CI] = 3.5 to 16, *P* < 0.001; Fig. 2). Similarly, territorials fathered 94/216 litters alone and were also significantly more likely to be found in multiple-sired than single-sired litters (odds ratio = 2.44, 95% CI = 1.5 to 4.1, *P* < 0.001; Fig. 2).

**Figure 3:**
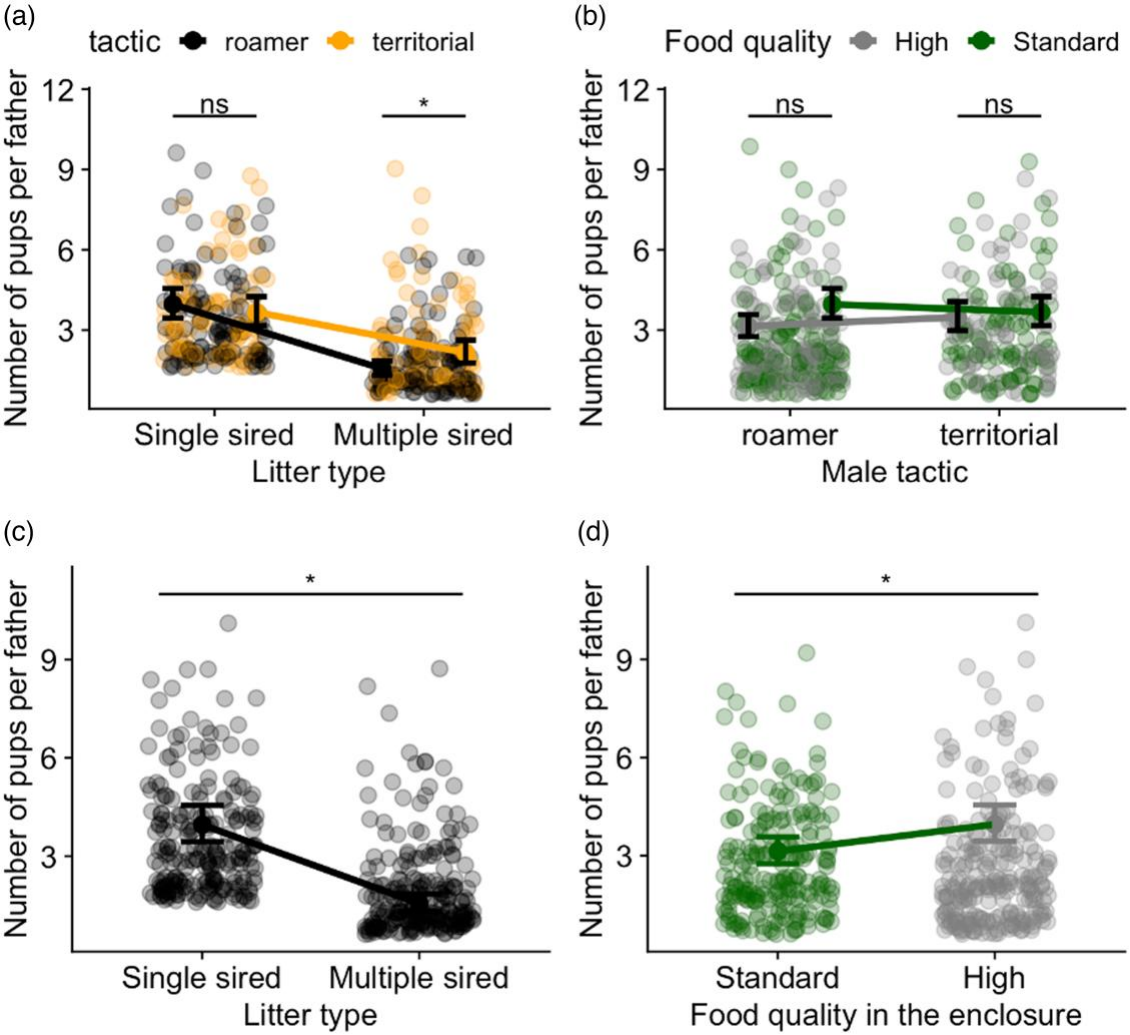
Predicted number of genotyped offspring per father across litter types and food quality. a) Raw data (jittered points) and model-predicted means (points ±95% CI) for territorial and roamer males in single- and multiple-sired litters. Territorial males sired significantly more offspring than roamers in multiple-sired litters, whereas no difference was observed in single-sired litters (*P* = 0.01). b) Model-predicted and raw counts of offspring per male by tactic and food quality enclosure. While the fitted model suggested a potential interaction between tactic and food quality, post hoc comparisons were not significant. c) Raw data and model-predicted offspring counts per male by litter type irrespective of tactic, showing that males sired fewer offspring in multiple-sired litters than in single-sired ones (*P* < 0.001). d) Raw data and predicted offspring counts per male by enclosure type irrespective of tactic, showing that males in standard-quality enclosures sired fewer offspring than in high-quality enclosures (*P* = 0.017).

We also inspected if the number of pups sired in each litter was predicted by the interaction between male tactic, multiple paternity and food quality in the environment without considering the relative contribution of each male, ie focusing on the differences between tactics as a function of environmental quality. Specifically, while territorials did not differ from roamers in the total number of pups sired in single-sired litters (*β* = −0.08, SE = 0.10, *P* > 0.1; Table 1), in multiple-sired litters, territorials sired significantly more pups compared with roamers (tactic × litter status: *β* = 0.40, SE = 0.17, *P* = 0.017; Fig. 3a; Table 1). In other words, territorial males fathered more pups even when they “experienced paternity loss.” Moreover, while the fitted model showed a difference in the offspring sired as a function of tactic and food quality (tactic × food quality), this was not confirmed in the post hoc test (Fig. 3b). As expected, each male sired fewer pups in multiple-sired litters than in single-sired ones (*β* = −0.93, SE = 0.11, *P* < 0.001; Fig. 3c; Table 1). The average number of pups males on a standard-quality food environment contributed was, again as expected, lower than that of males on a high-quality one (*β* = −0.23, SE = 0.10, *P* = 0.017; Fig. 3d; Table 1).

**Figure 4:**
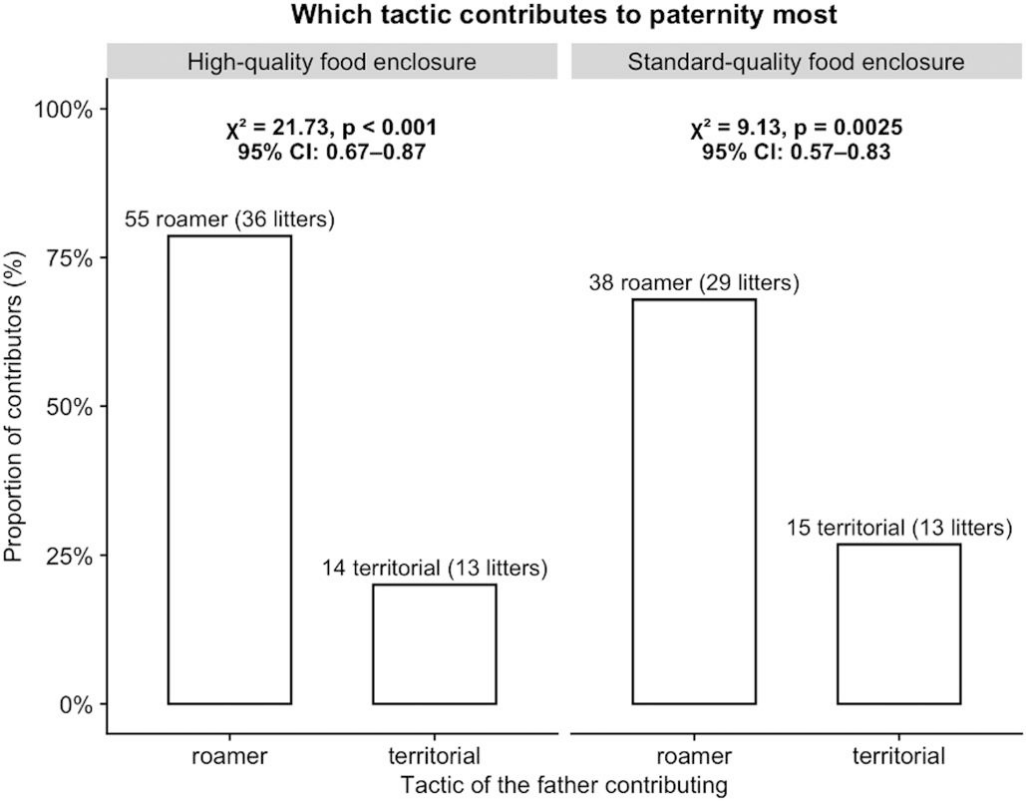
Proportion of contributing fathers by tactic per food quality enclosure. Bars show the proportion of males that contributed to paternity (ie sired offspring in litters where another male was the main father) according to their tactic at conception. Numbers above bars indicate the number of fathers and the number of litters they contributed to. A one-sample proportion test (continuity corrected) revealed that most were roamers, ie more than expected by chance.

We last quantified how many pups were sired by each tactic in multiple-sired litters (*n* = 74), after excluding the pups of the male with the highest contribution, ie the “main father.” Tactics differed in their relative contribution within, but not across, food quality enclosures. In the high-quality food enclosures, roamers contributed in 36 of the stolen litters (∼78.6% of those steal events) in 55 such events in total; and averaged 1.76 pups per litter. In contrast, territorials contributed to paternity in 13 litters (∼20.0%; 14 events in total), averaging 1.29 pups per litter (remainder 1.4% were unassigned). Similarly, in the standard-quality enclosures, roamers contributed to paternity 38 times in 29 litters (∼67.9%), averaging 2.39 pups per litter; while territorials 15 times in 13 litters (∼26.8%), averaging 1.60 pups per litter (5.3% of cases unassigned). The difference in tactic contribution within food quality enclosures was statistically significant (*χ*^2^ test: *P* < 0.001; Fig. 4).

## Discussion

Our study shows a positive correlation between the number of roamers, but not of territorials, around conception and the incidence of multiple paternity, indicating that roamers indeed pursue sneaky matings and drive variation in litter composition. Confirming this, roamers were present in multiple-sired litters roughly twice as often as territorials, which (ie territorials) nevertheless still sired most offspring (in multiple-sired litters). Our study also shows that under poorer environmental conditions, specifically in an environment with relatively worse food quality, the average male reproductive success declines regardless of reproductive tactic; while the incidence of multiple paternity increases in larger litters. Overall, we extend previous work showing that female reproductive outcomes are shaped not only by social dynamics (Say et al. 1999; Zamudio and Sinervo 2000) but also by fine-grained variation in male reproductive dynamics, revealing an interaction between male ARTs and female polyandry. As such, the persistence of ARTs may ultimately depend on female mating behavior, with polyandry enabling different strategies to achieve reproductive success (Neff and Svensson 2013).

### The link between male ARTs, polyandry and multiple paternity

We found that variation in the incidence of multiple paternity was predicted by an increasing number of roamers, but not of territorials, present around conception. This suggests that while territorials sire most pups and exhibit higher reproductive success (Taborsky et al. 2008; Da Silva et al. 2025; Darmis et al. 2025), the abundance of roamers is linked to multiple paternity, possibly because roamers sneak matings. Similar, albeit indirect, patterns have been reported in other taxa. For example, in orb-weaving spiders (*Trichonephila clavipes*), males prolong mate guarding when satellite males are abundant (Matto et al. 2021), possibly because the latter attempt to steal matings. Likewise, in ground squirrels (*Callospermophilus lateralis*), multiple paternity increases linearly with male density, suggesting that males cannot effectively monopolize females when male–male competition elevates (Wells et al. 2017). In contrast, observations in striped mice indicate that territorial males steal most paternity from other territorials (Schradin et al. 2010), highlighting species-specific differences in reproductive dynamics.

Here and in other systems, several non-mutually exclusive mechanisms may explain how roamers achieve matings: they may actively pursue copulations, females may preferentially mate with them, or both. First, the most parsimonious explanation is that roamers do the “best-of-a-bad-job” by adopting a tactic that reduces the risk of remaining unmated (Dawkins 1980; Gross 1996). Under this view, polyandry and the resulting multiple paternity may still be beneficial for females, for example by reducing the risk of infanticide through diluted paternity certainty (Dean et al. 2006; Solomon and Keane 2008; Klemme and Ylönen 2010). Alternatively, multiple mating may be female-driven if females actively mate multiply to reduce the likelihood of reproducing solely with a familiar or related male (Waser and De Woody 2006). In this case, multiple mating is not specifically directed toward roamers; rather, roamers may disproportionately gain access to matings because they are more abundant and rely on roaming tactics that increase mating encounters. Finally, ecological constraints may also contribute. Mate-guarding can be less effective under certain conditions (Rubenstein 2007), for example when female availability is low, leading most males to adopt a roaming tactic in order to locate mating opportunities and to reduce their relative investment in mate-guarding.

Overall, our results extend previous work on polyandry in rodents (Klemme et al. 2006; Firman and Simmons 2008a, 2008b; Klemme and Ylönen 2010; Fickel and Weyrich 2011; Thonhauser, Raveh, et al. 2014; Thonhauser, Thoß, et al. 2014; Darmis and Guenther 2025) indicating that multiple paternity arises from interactions between male and female behavior rather than from either sex alone. Similar interaction-driven patterns have been reported for extra-pair paternity in birds, sometimes involving male and female age effects (Ramos et al. 2014; Michálková et al. 2019).

### Which tactic steals paternity?

We found that variation in paternity arises primarily from differences in the abundance of male ARTs. Specifically, the positive association between roamers with multiple paternity we reported above was further supported by direct evidence that roamers act as the main “sneaking” tactic, being disproportionately present (3 times more) in multiple-sired litters compared with territorials (odds ratio: 7.2 versus 2.4, respectively).

To our knowledge, these results are the first clear demonstration, in a mammalian polygynandrous system, that male ARTs and female polyandrous mating interact. Our findings align with theoretical expectations that polyandry plays a central role in shaping the possible evolution and maintenance of male ARTs (Neff and Svensson 2013). Indeed, ARTs and the outcomes of polyandry (ie here multiple paternity) might be tightly coupled through feedback dynamics: increased polyandry can intensify selection for sneaking tactics, while the resulting variation in male reproductive success (ie from polyandry) may in turn reinforce the maintenance of ARTs. In particular, roamers can gain paternity through sneaking when territorial males fail to fully monopolize matings, either because females mate multiply with several males or because sneakers successfully force copulations. Regardless of the underlying mechanism, roamers achieve reproductive success. This generates a situation in which reproductive success depends not only on competitive ability per se, but also on the interaction between male tactics, female mating behavior and ecological constraints on monopolization. Thus, the success of roamers should not be viewed as an independent strategy, but rather as an emergent outcome of incomplete mate monopolization and female mating decisions. Importantly, this framework highlights a key condition: for polyandry to facilitate the evolution and maintenance of ARTs, it must generate variance in reproductive success among tactics, ie tactic-specific multiple paternity (or, in other systems, extra-pair or extra-group paternity).

### The role of environmental conditions on the link between ARTs and polyandry

Our study advances our understanding of how environmental conditions shape mating patterns by integrating 4 key components: (i) male ARTs, (ii) litter status (single versus multiple sired), (iii) the fitness outcomes associated with multiple paternity per tactic, and (iv) environmental quality. We show that, although the fitness benefits of multiple paternity are context-dependent (Darmis and Guenther 2025), the relative reproductive success of male ARTs does not appear to shift with environmental quality, at least for the part of males’ life that we studied and with respect to the number of offspring that survive to weaning. While this was the opposite of what we predicted, it is possible that such effects will be detectable when competition intensifies or when environmental conditions change abruptly within populations, which was not the case here. Moreover, it may be that environmental quality primarily affects the survival of ARTs rather than their reproductive success.

We found that larger litters in standard-quality food environments were more likely to be multiple sired, consistent with previous work showing context-dependent effects of polyandry (Darmis and Guenther 2025). One possible explanation is that lower-quality (harsher in general) environments reduce overall individual fitness and intensify male–male competition for limited mating opportunities, leading to increased paternity loss by dominant males. Alternatively, such conditions may increase the importance of female choice and thereby elevate rates of polyandry and multiple paternity, for example if females engage in additional matings with diverse males as a form of bet-hedging to improve offspring fitness (Darmis and Guenther 2025). Distinguishing between these mechanisms was beyond the scope of our analyses and remains an important direction for future work. Overall, our results, coupled with other studies on the context-dependency of polyandry (Arct et al. 2013; Maldonado-Chaparro et al. 2018; Yang et al. 2024; Darmis and Guenther 2025), indicate that the drivers and consequences of polyandry might arise from complex interactions between environmental and social conditions simultaneously.

### Concluding remarks

Although we did not account for solitary or communal breeding (Ferrari et al. 2019; Ferrari et al. 2022), which is known to be linked to polyandry (Auclair et al. 2014), our results suggest that polyandry is closely linked to variation in the male reproductive landscape, consistent with patterns reported elsewhere (Lichter et al. 2020). Future work should disentangle whether male ARTs are maintained by female preferences for alternative male phenotypes, and whether such preferences can generate the conditions under which ARTs evolve (Neff and Svensson 2013; Pizzari and Wedell 2013). From an evolutionary perspective, our results point to a key conclusion: the evolution and maintenance of polyandry, that is its drivers and fitness consequences, are shaped by interacting variation in both the social (as we show here) and ecological environment (as shown previously: Darmis and Guenther 2025). Together, these factors determine the balance between costs and benefits of multiple mating and thereby the evolutionary stability of both male and female mating behavior.

## Data Availability

Analyses reported in this article can be reproduced using the data provided by Darmis and Guenther (2026).
